# Supplementary material for: Edge-Rich Interconnected Graphene Mesh Electrode with High Electrochemical Reactivity Applicable for Glucose Detection
Source: Nanomaterials (Basel). 2021 Feb 17;11(2):511. doi: 10.3390/nano11020511 (PMC7922656; doi:10.3390/nano11020511)
Supplement: Supplementary file 1 [file nanomaterials-11-00511-s001.pdf]

nanomaterials

Supplementary Materials

Edge-Rich Interconnected Graphene Mesh Electrode with High Electrochemical Reactivity Applicable for Glucose Detection

Van Viet Tran [1], Duc Dung Nguyen [1,2], Mario Hofmann [3], Ya-Ping Hsieh [4], Hung-Chih Kan [1] and Chia-Chen Hsu [1,*]

1 Department of Physics, National Chung Cheng University, Chiayi 621, Taiwan; viettran.apc@gmail.com (V.V.T.); ddnguyen161@gmail.com (D.D.N.); phyhck@ccu.edu.tw (H.-C.K.)
2 Center for High Technology Development, Vietnam Academy of Science and Technology, Hanoi100000, Vietnam
3 Department of Physics, National Taiwan University, Taipei 10617, Taiwan; mario@phys.ntu.edu.tw (M.H.)
4 Institute of Atomic and Molecular Sciences, Academia Sinica, Taipei 106, Taiwan; yphsieh@gate.sinica.edu.tw (Y.-P.H.)
* Correspondence: phycch@ccu.edu.tw (C.-C.H); Tel.: +886-5-272-0411 (ext. 66305)

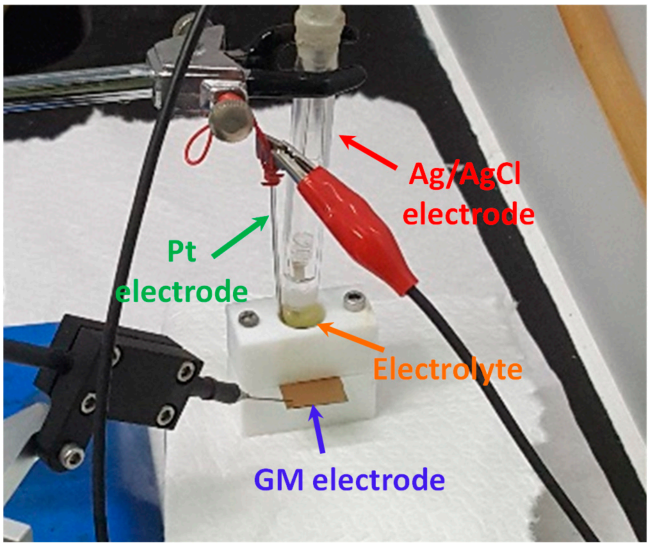

Figure S1. Cyclic voltammetry measurement setup

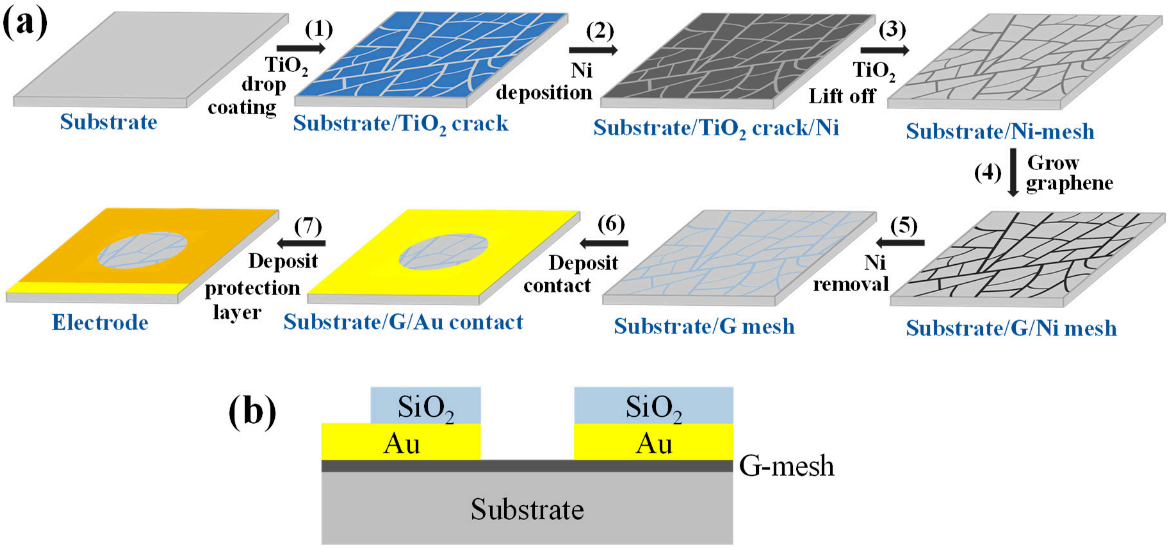

Figure S2. (a) Schematic of fabrication procedure of the GM electrode. (b) The cross-section view of the electrode.

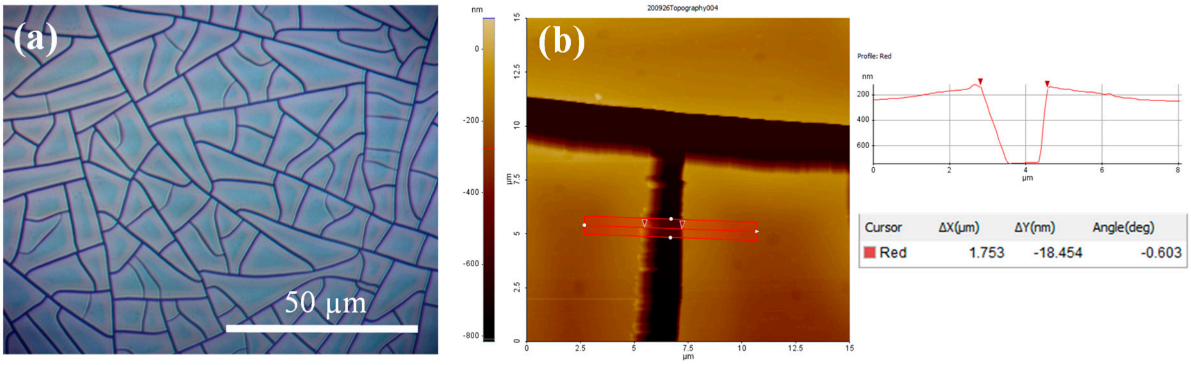

Figure S3. (a) Optical microscopic image and (b) AFM profile of representative cracks of the TiO_2 template V-50.

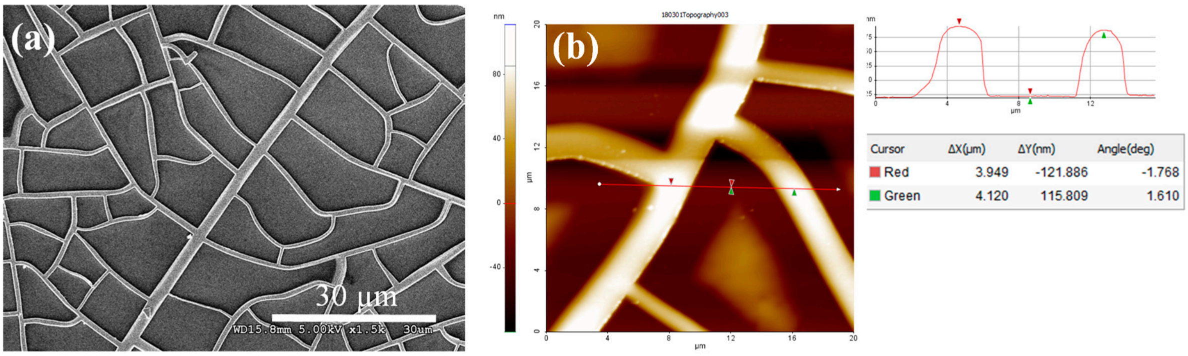

Figure S4. (a) SEM image of the Ni mesh obtained with the TiO_2 template V-50. (b) AFM profile of the NM V-50.

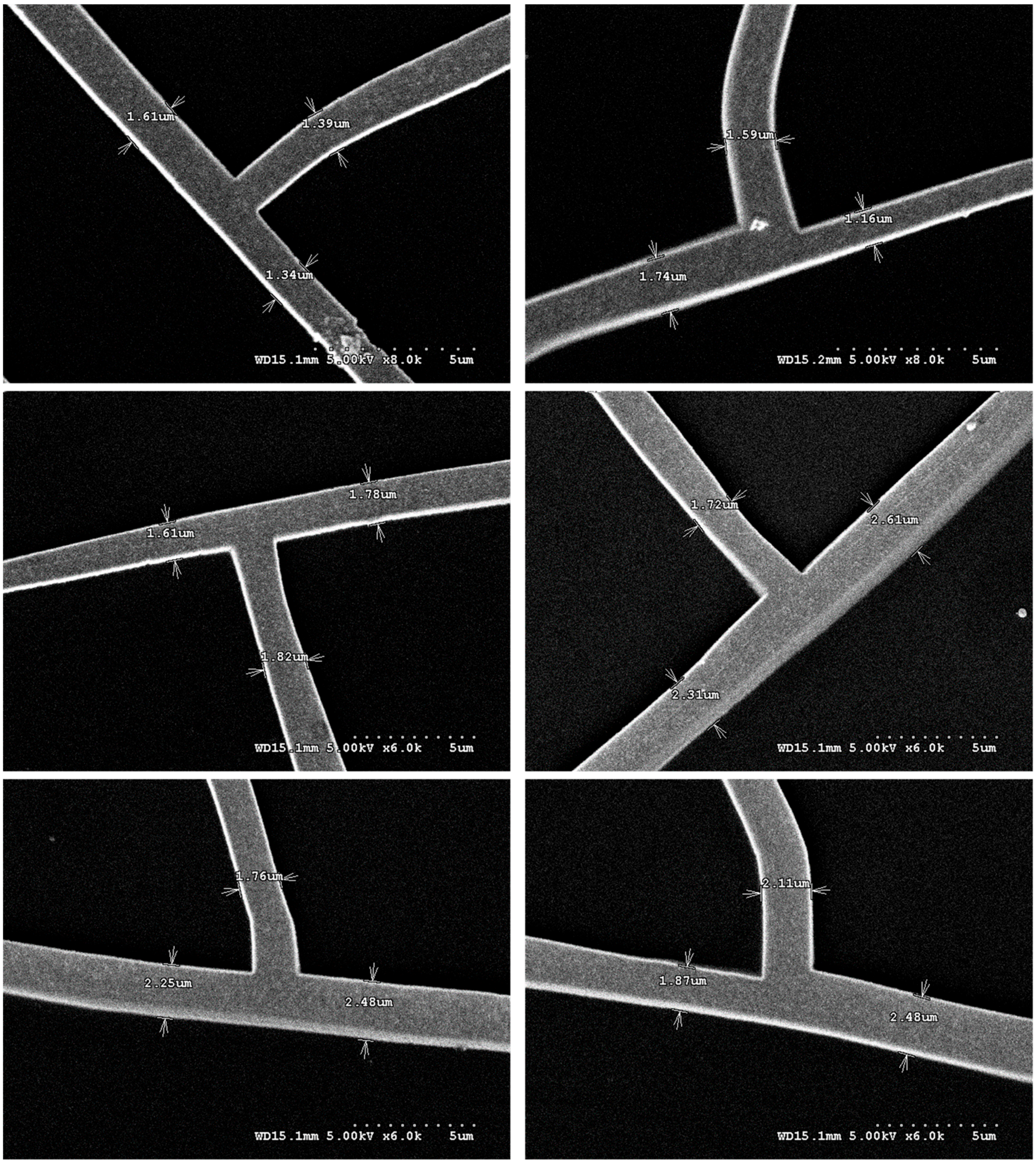

Figure S5. The SEM images of several random positions of the Ni mesh (NM V-50) sample obtained from the TiO_2 template V-50.

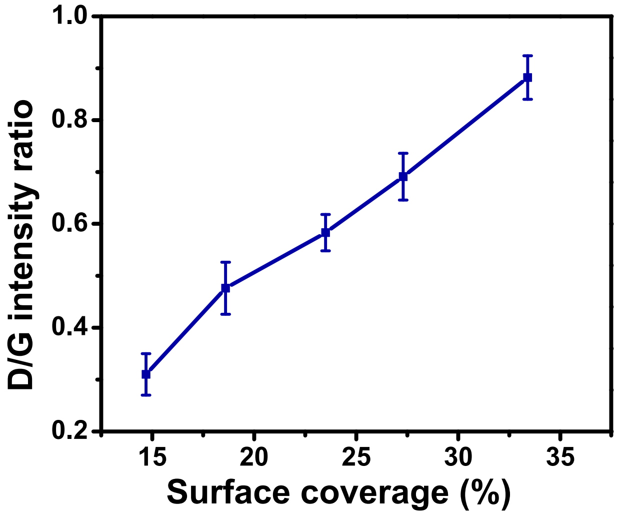

Figure S6. The intensity ratio of D/G peak vs. surface coverage of graphene wires of each GM electrode.

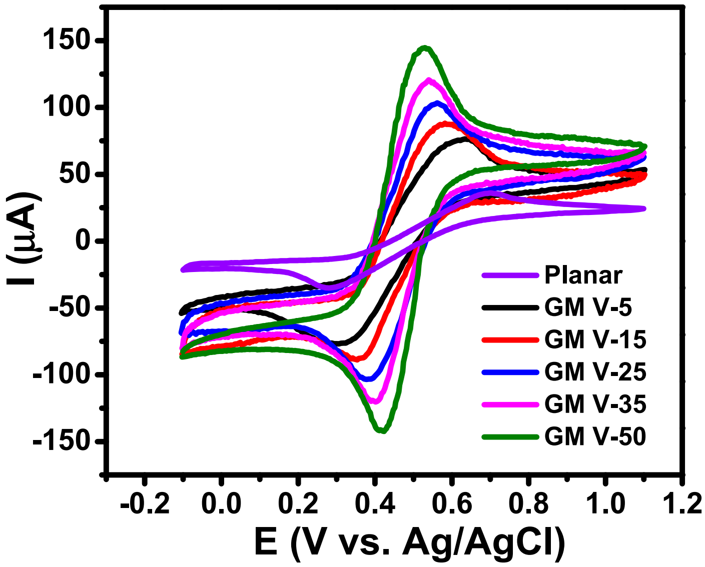

Figure S7. Representative cyclic voltammograms of graphene electrodes with different SC of graphene wire in 1 mM ferrocene and 0.1 M Bu_4NPF_6 in acetonitrile electrolyte obtained at a scan rate of 0.02 V/s.

Table S1. Average width and average edge length of graphene wire

Sample	GM V-5	GM V-15	GM V-25	GM V-35	GM V-50
Average width (µm)	1.98	1.93	1.85	1.89	1.87
Average edge length (m)	5.48	9.31	13.67	17.94	24.04

The edge length is determined from Equation S1:

$$edge\ length = \frac{A \times surface\ coverage}{wire\ width} \times 2 \quad \text{(Equation S1)}$$

Where: A is the electroactive area of electrodes

1. Comparison of the CV Diagram of planar Graphene and Graphene Mesh

For comparison, the CV of a planar graphene electrode was measured under the same conditions as the graphene mesh samples. The planar graphene was grown atop of a 120 nm thick Ni thin film deposited on a SiO_2/Si substrate with the same fabrication conditions as the GMs, and then immersed into $FeCl_3$ (0.5 M) solution for overnight to remove the interlayer Ni thin film. Figure S8a exhibits the optical microscope image of the planar graphene. Since the planar graphene was synthesized by the direct transfer growth method, its surface morphology replicated that of the Ni surface, whose roughness drastically increased during thermal annealing process. Consequently, the surface morphology of the planar graphene was not smooth [1]. From the Raman spectrum of the planar graphene shown in Figure S8b, the I_{2D}/I_G is ~ 0.62, close to those of the GM samples, representing that few-layer of graphene was formed. The I_D/I_G is ~ 0.125 which is smaller than those of GM samples, suggesting that the planar graphene possesses lower density of defects which is mainly attributed to the removal of Ni. As displayed in Figure S7, compared with GM electrodes, the planar graphene electrode exhibits a lower current intensity and larger peak potential separation, indicating much weaker EC reactivity of the planar graphene electrode [2, 3] (as presented by the k values).

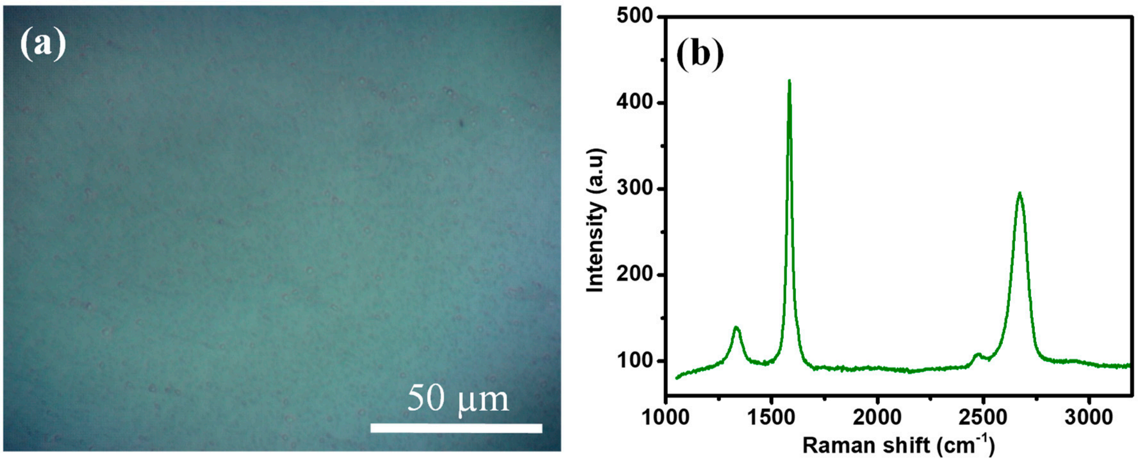

Figure S8. Planar graphene. (a) OM image, (b) Raman shift spectrum.

2. Calculation of the Transfer Coefficient

The transfer coefficient (α) can be determined from plots of scan rate (ν) versus anodic peak position $E_{p,a} = f(\log \nu)$ (as displayed in Figure S9) in which yields straight lines with a slope equal to

$2.3RT/(1\text{-}\alpha)nF$ [4]. Where: R (= 8.314 J·mol^{-1}·K^{-1}) is gas constant, T is room temperature (K), n is the number of electrons per molecule and F (= 96485.333 C·mol^{-1}). The values of α are shown in Table S2.

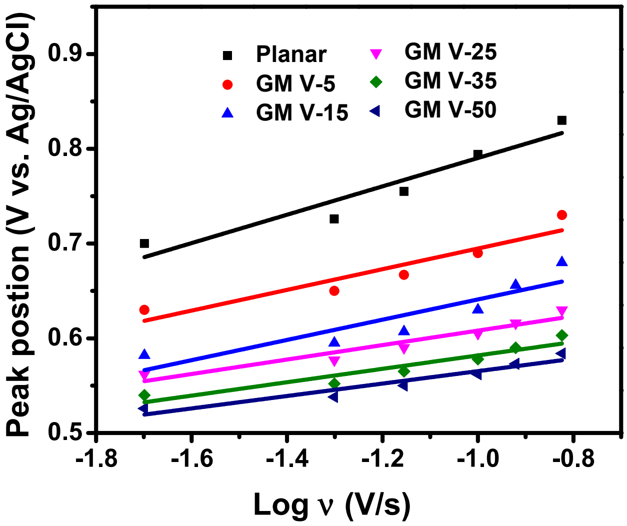

Figure S9. Anodic peak position vs log(scan rate).

Table S2. The transfer coefficients (α) between electroactive compound and the graphene electrode.

Sample	Planar	GM V-5	GM V-15	GM V-25	GM V-35	GM V-50
Transfer coefficient	0.07	0.0668	0.0666	0.0644	0.064	0.0636

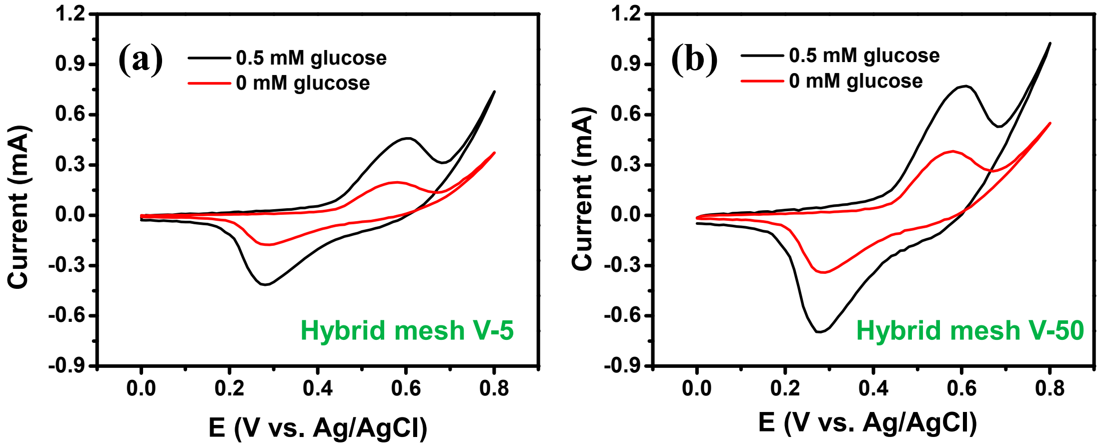

Figure S10. Cyclic voltammograms of (a) the hybrid mesh V-5 and (b) V-50 electrodes in 0.1 M NaOH with/without glucose. (scan rate: 50 mV·s^{-1}).

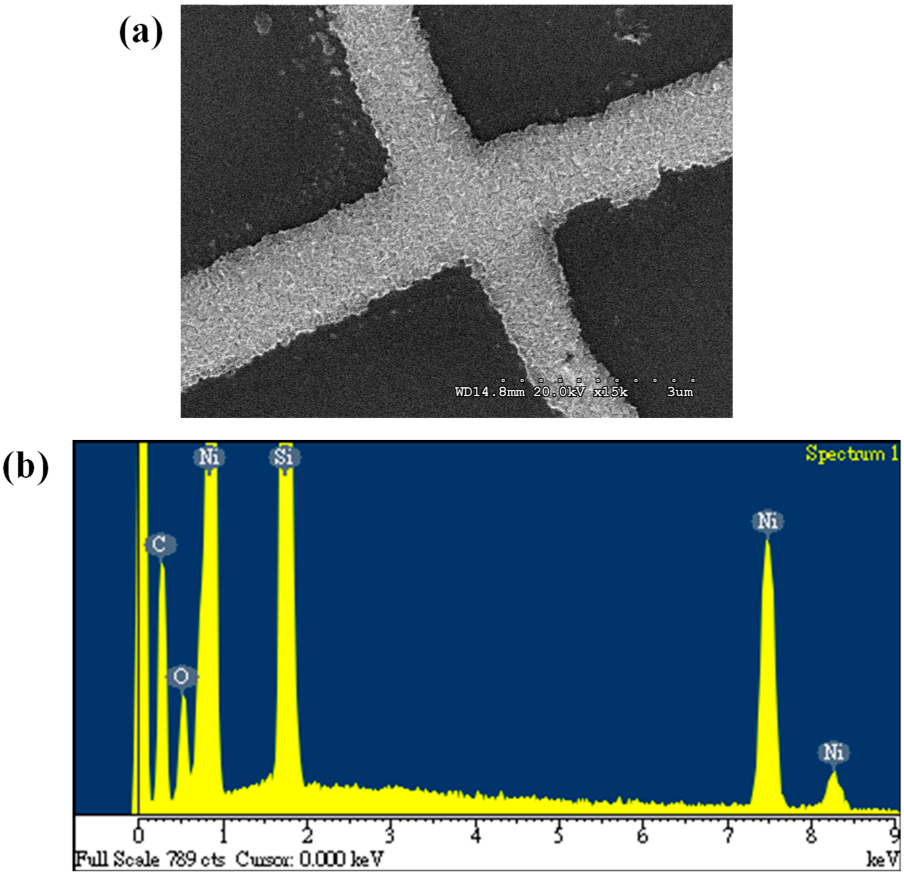

Figure 11. (a) SEM image of the graphene/Ni hybrid mesh obtained with the TiO_2 template V-50; (b) EDS analysis of the graphene/Ni hybrid mesh on SiO_2/Si substrate.

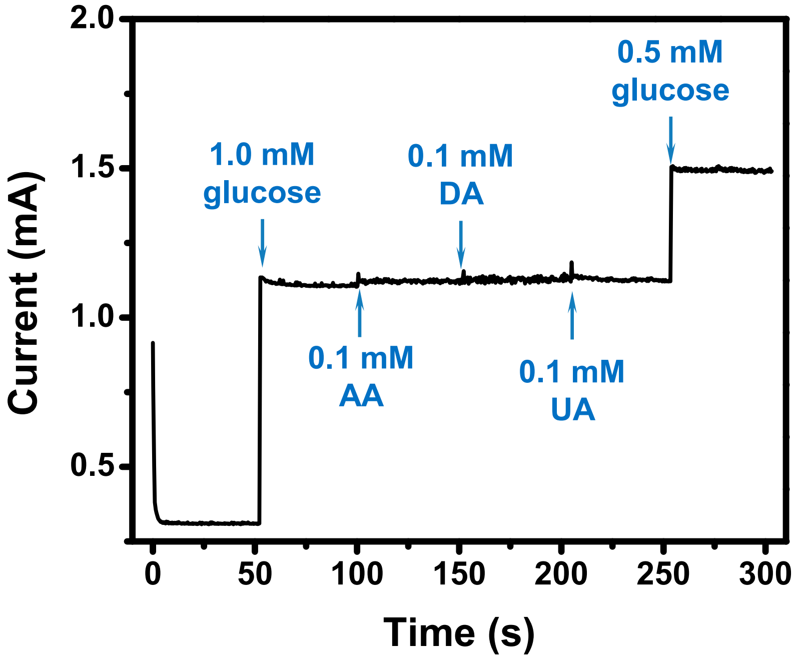

Figure 12. Selectivity of the graphene/Ni hybrid mesh V-50 measured with 1 mM glucose, 0.1 mM AA, 0.1 mM DA, 0.1 mM UA and 0.5 mM glucose in 0.1 M NaOH.

References

1. Pan, G.; Li, B.; Heath, M.; Horsell, D.; Wears, M.L.; Taan, L.A.; Awan, S. Transfer-free growth of graphene on siO_2 insulator substrate from sputtered carbon and nickel Films. *Carbon* **2013**, *65*, 349–358.
2. Yuan, W.; Zhou, Y.; Li, Y.; Li, C.; Peng, H.; Zhang, J.; Liu, Z.; Dai, L.; Shi, G. The edge- and basal-plane-specific electrochemistry of a single-layer graphene sheet. *Sci. Rep.* **2013**, *3*, 2248.
3. Li, K.; Jiang, J.; Dong, Z.; Luo, H.; Qu, L. A linear graphene edge nanoelectrode. *Chem. Commun.* **2015**, *51*, 8765.
4. Laviron, E. General expression of the linear potential sweep voltammogram in the case of diffusionless electrochemical systems. *J. Electroanal. Chem.* **1979**, *101*, 19-28.
